# Selective Cerebrospinal Fluid Hypothermia: Bioengineering Development and In Vivo Study of an Intraventricular Cooling Device (V-COOL)

**DOI:** 10.1007/s13311-022-01302-y

**Published:** 2022-09-21

**Authors:** Simone Beretta, Alessandro Versace, Gianfranco Fiore, Marco Piola, Beatrice Martini, Vittorio Bigiogera, Lorenzo Coppadoro, Jacopo Mariani, Lorenzo Tinti, Silvia Pirovano, Laura Monza, Davide Carone, Matteo Riva, Giada Padovano, Gilda Galbiati, Francesco Santangelo, Marco Rasponi, Francesco Padelli, Isabella Giachetti, Domenico Aquino, Susanna Diamanti, Laura Librizzi, Maria Grazia Bruzzone, Marco De Curtis, Carlo Giussani, Erik P. Sganzerla, Carlo Ferrarese

**Affiliations:** 1 Laboratory of Experimental Stroke Research, School of Medicine and Surgery, University of Milano Bicocca, Via Cadore 48, 20900 Monza, Italy; 2 Department of Neuroscience, San Gerardo Hospital, ASST Monza, Monza, Italy; 3 Department of Electronic, Information and Bioengineering, Politecnico Di Milano, Milan, Italy; 4 Department of Diagnostics and Technology, Fondazione IRCCS Istituto Neurologico Carlo Besta, Milan, Italy; 5 Neuroradiology Unit, Fondazione IRCCS Istituto Neurologico Carlo Besta, Milan, Italy

**Keywords:** Hypothermia, Device, Cerebrospinal fluid, Vasospasm, Cerebral ischemia, Neuroprotection

## Abstract

Hypothermia is a promising therapeutic strategy for severe vasospasm and other types of non-thrombotic cerebral ischemia, but its clinical application is limited by significant systemic side effects. We aimed to develop an intraventricular device for the controlled cooling of the cerebrospinal fluid, to produce a targeted hypothermia in the affected cerebral hemisphere with a minimal effect on systemic temperature. An intraventricular cooling device (acronym: V-COOL) was developed by in silico modelling, in vitro testing, and in vivo proof-of-concept application in healthy Wistar rats (*n* = 42). Cerebral cortical temperature, rectal temperature, and intracranial pressure were monitored at increasing flow rate (0.2 to 0.8 mL/min) and duration of application (10 to 60 min). Survival, neurological outcome, and MRI volumetric analysis of the ventricular system were assessed during the first 24 h. The V-COOL prototyping was designed to minimize extra-cranial heat transfer and intra-cranial pressure load. In vivo application of the V-COOL device produced a flow rate-dependent decrease in cerebral cortical temperature, without affecting systemic temperature. The target degree of cerebral cooling (− 3.0 °C) was obtained in 4.48 min at the flow rate of 0.4 mL/min, without significant changes in intracranial pressure. Survival and neurological outcome at 24 h showed no significant difference compared to sham-treated rats. MRI study showed a transient dilation of the ventricular system (+ 38%) in a subset of animals. The V-COOL technology provides an effective, rapid, selective, and safe cerebral cooling to a clinically relevant degree of − 3.0 °C.

**Supplementary Information:**

The online version contains supplementary material available at 10.1007/s13311-022-01302-y.

## Introduction

Although recanalization therapies have greatly improved outcome of patients with acute ischemic stroke [1], treatment of other types of cerebral ischemia, which are not caused by a focal thrombotic occlusion, remains largely unsatisfactory. In particular, delayed cerebral ischemia (DCI) associated with severe vasospasm accounts for up to 30% of mortality and morbidity after aneurysmal subarachnoid hemorrhage (SAH) and its management remains unsettled [2]. Other examples of non-thrombotic cerebral ischemia are intraoperative hemodynamic stroke [3] and severe reversible cerebral vasoconstriction syndrome [4]. Hypothermia is a highly promising neuroprotective strategy with consistent anti-ischemic activity in different animal models [5], pleiotropic mechanisms of action [6], and potential for combination therapy [7]. Moreover, hypothermia could be potentially beneficial in other non-ischemic acute brain disorders, such as traumatic brain injury [8] and refractory status epilepticus [9]. A major limitation for the implementation of systemic hypothermia in neurological and neurosurgical patients is that cooling to a core temperature of 34 °C, like in most animal studies, requires prolonged sedation and mechanical ventilation [10]. Moreover, systemic hypothermia is associated with potentially severe cardiac, infectious, and hematologic side effects, which are dependent on its depth and duration [11].

Selective cerebrospinal fluid (CSF) hypothermia is a new unexplored concept, which may provide the following major advantages, compared to systemic hypothermia: (i) controlled cooling of the affected hemisphere; (ii) deeper degree and prolonged duration of controlled hypothermia; (iii) reduction of systemic side effects; and (iv) maintaining the patient awake and accessible for neurological examination. In this study, we developed a new intraventricular cooling device (acronym: V-COOL) for controlled cooling of the CSF, starting with a bioengineering approach for analytical and in vitro modelling and progressing to proof-of-principle in vivo experiments in rats.

## Methods

### Analytical Modelling of the V-COOL Device and In Vitro Preliminary Evaluation

A simplified, mono-compartmental model describing the thermal balance of brain cooling by means of the extracorporeal recirculation of CSF was considered. The balance involves the internal energy transferred to/from the brain tissue by blood flow, the heat produced by parenchymal brain metabolism, and the internal energy transfer induced by the extracorporeal circulation of CSF. Before initiating the treatment, the brain tissue may be considered at steady state, with a temperature constant in time. As the treatment starts, cool CSF is injected into the ventricular cavity, while warm CSF is withdrawn at an equal flow rate, thus generating a thermal unbalance aimed at reducing brain temperature. Under some reasonable simplifying assumptions (e.g., ideal heat exchange between the CSF and brain parenchyma, uniform specific heat *c*, and uniform density *ρ*), the following time course of brain temperature is yielded:

$$T={T}_{\infty }-\left({T}_{\infty }-{T}_{0}\right){e}^{-t/\tau }$$

where *T*_0_ is the pre-treatment temperature, *T*_∞_ is the final settling temperature, and *τ* is the time constant of the process (99% settling time equals 5$\tau$). These quantities, in turn, depend upon the treatment parameters as:

$$\tau =\frac{{V}_{t}}{{Q}_{b}+{Q}_{CSF}}\; ;\qquad {T}_{\infty }=\frac{{Q}_{b}{T}_{bi}+{Q}_{CSF}{T}_{CSFi}+\Phi /\rho c}{{Q}_{b}+{Q}_{CSF}}$$

where subscripts *b* and *CSF* refer to blood and cerebrospinal fluid, respectively; *Q* are flow rates, *T*_*i*_ are inlet fluid temperatures, *V*_*t*_ is the total intracranial volume, and Φ is the heat produced by metabolism.

A simplified phantom model was developed for an in vitro preliminary feasibility evaluation of the experimental setting. The brain tissue was modelled as a spherical volume of water of 2 mL, surrounded by a transparent, heat-insulating polymethylmethacrylate (PMMA) capsule. Temperature within the sphere was measured by a thermocouple probe deepened into the fluid, replicating the simulated parenchymal temperature. Two holes in the PMMA wall allowed for water at 37 °C to be injected/withdrawn with a peristaltic pump, thus replicating blood perfusion at a fixed 2.2 mL/min flow. A separate hole in the wall allowed a dual-lumen V-COOL cannula tip to be deepened into the core fluid volume. Water at 15 °C was injected through the inflow lumen of this cannula with a syringe pump and, simultaneously, withdrawn at the same flow rate (0.5, 0.8, 1.0 mL/min) through the outflow lumen, thus representing the circulation of cool CSF.

### In Vivo Prototyping of the V-COOL Device

**Fig. 1:**
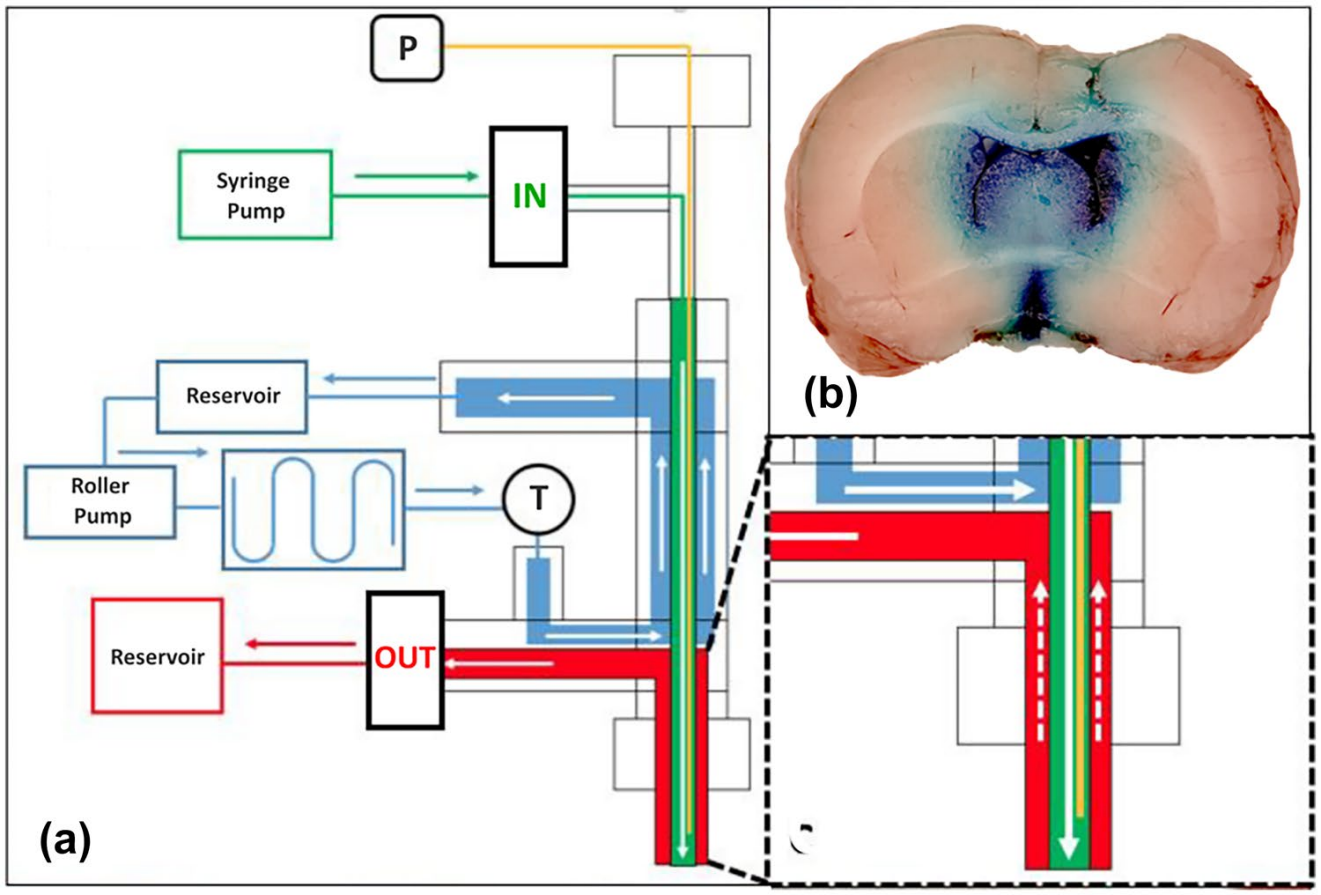
In vivo prototyping of the V-COOL device (**a**). Successful intraventricular access, verified by injection of methylene blue in the inner lumen of the cannula (**b**). IN inflow line, OUT outflow line, P inline pressure sensor, T temperature sensor

The selective CSF hypothermia principle relied upon automated extracranial circulation of the CSF. Specifically, the V-COOL device consisted of a fluidic apparatus providing for an open-loop circulation of mixed artificial CSF (aCSF) and native CSF. aCSF has been routinely used as a vehicle solution for repetitive administration of test agents to the central nervous system of laboratory animals, with no safety concerns [12]. The V-COOL device provides access to the frontal horn of the left lateral ventricle through a purposely developed concentric double-lumen cannula, suitable to be applied under stereotaxic guidance. Within an external hydraulic circuit, connected with the cannula, room temperature aCSF was slowly injected (0.2 to 0.8 mL/min) into the inner lumen of the cannula and gradually cooled down to 10 °C during the inflow process by heat transfer via a refrigerating jacket. The jacket, in turn, was recirculated with a saline solution, which was kept continuously cold via a refrigerating circuit based on the Peltier cell technology (Fig. 1a). The injected aCSF mixed with the native CSF, thus generating a net cooling effect of the CSF compartment. The fluid flushed out by passive outflow via the outer lumen of the cannula, into a waste iso-pressure reservoir. In all experiments, with the exception of those assessing functional outcome at 24 h, successful intraventricular access was verified by gentle injection of methylene blue in the inner lumen of the cannula at the end of the experiment (Fig. 1b).

### In Vivo Study Design, Animals, and Surgery

The experimental protocol was conducted in accordance with the national and European guidelines on the use of laboratory animals (D.L. 26/2014; 2010/63/EU), received a project license for Animal Care and Use from the Italian Ministry of Health (81/2015-PR). Consecutive rats were included. The primary outcome was net cerebral cooling. A sample size of 38 animals was estimated to detect a 1.5 °C difference in cerebral temperature across three V-COOL flow rates, with 0.80 power and 0.05 type I error. Considering the exploratory purpose of the study and logistic constraints, randomization and blinded outcome assessment were not applied.

Adult male Wistar rats (weight 282 ± 32 g; total *n* = 42) were anesthetized with intraperitoneal injection of ketamine (90 mg/kg) and xylazine (10 mg/kg). Rats were placed on the brain stereotaxic apparatus in prone position. A midline incision was made in the scalp and a burr hole was made to slowly insert the distal portion of the V-COOL device in the frontal horn of the left lateral ventricle at the cranial coordinates ML − 1.55, AP − 0.80, and DV 4.23, related to bregma. The V-COOL device was activated for the desired duration (10 to 60 min). After the removal of the V-COOL device, scalp was sutured and rats were placed in single cages, where they recovered from anesthesia and had free access to food and water. Temperature was maintained at 37 °C using a heating pat from the onset of ketamine-xylazine anesthesia until the activation of the V-COOL device, then external heating was stopped.

### Cerebral and Systemic Temperature Monitoring

Cerebral cortical temperature was continuously monitored using a fiber optic temperature probe (OTG-M420, Opens, Canada) connected to a single-channel signal conditioner (PicoM, Opsens, Canada), gently introduced in the left (ipsilateral) parietal lobe cortex via a burr hole at the cranial coordinates ML − 4.00, AP − 4.16, and DV 1.13. Systemic temperature was continuously monitored using a rectal temperature probe (Ugo Basile, Italy). Both cerebral and systemic temperature monitoring started before the insertion of the V-COOL device and lasted for approximately 5 min after the device was switched off. The net cerebral cooling, $\Delta T(t)$, related to a certain time interval was expressed correcting the measured change in cerebral temperature by the measured change in the systemic temperature, according to the following formula:

$$\Delta T\left(t\right)=\left({T}_{c}\left(0\right)-{T}_{c}(t)\right)-\left({T}_{r}\left(0\right)-{T}_{r}(t)\right)$$

where ${T}_{c}\left(0\right)$ is the baseline cortical temperature; *T*_*c*_(*t*) is the cortical temperature at the current time, *t*; *T*_*r*_(0) is the baseline rectal temperature; and *T*_*r*_(*t*) is the rectal temperature at time *t*.

In a further set of preliminary experiments (*n* = 4), a second temperature probe was introduced in the right (contralateral) parietal lobe cortex via a burr hole at the cranial coordinates ML + 4.00, AP − 4.16, and DV 1.13.

### Invasive Intracranial Pressure Monitoring

In a subset of animals, direct invasive intracranial pressure (ICP) monitoring was performed using a fiber optic pressure sensor (OPP-M250, Opsens, Canada) connected to a single-channel signal conditioner (LifeSens, Opsens, Canada), gently introduced in the same burr hole used for cortical temperature measurement. ICP measurements were recorded with a frequency of 20 Hz using a dedicated software, then they were exported for analysis.

### Neurologic Outcome Assessment

A subset of animals treated with V-COOL were compared to sham-operated animals that received anesthesia, craniotomy, and insertion of the V-COOL device, leaving it switched off. Mortality was assessed 24 h after V-COOL application. Garcia et al. neuroscore [13] was used to assess global neurological functioning at 24 h and was expressed as a dichotomous variable as follows: “good functional outcome” (scores 14 to 18) or “poor functional outcome” (scores 3 to 13). Corner turning test [14] was used to score sensorimotor asymmetries and visual-spatial deficits and was expressed as a dichotomous variable as follows: “good functional outcome” (scores > 30% right turns) or “poor functional outcome” (scores ≤ 30%). Ordinal data for Garcia neuroscore (3 to 18) and continuous data for corner turning test (0 to 100%) were also shown.

### MRI-Based Volumetric Analysis of the Ventricular System

Rats were placed in a horizontal-bore small animal 7 T MRI scanner (BioSpec 70/20 USR, Bruker, Ettlingen, Germany), equipped with a rat brain dedicated surface radiofrequency coil used as signal receiver with a 72-mm-diameter volume resonator. A Rapid Acquisition with Refocused Echoes (RARE) T2-weighted coronal sequence (TR = 3750 ms; TE = 33 ms; FA = 90°/180°; RARE factor = 8; FOV = 30 × 24 mm^2^; in-plane resolution 0.150 × 0.150 mm^2^, 30 continuous slices, slice thickness 0.500 mm; number of averages 10; time duration 14 min) under isoflurane anesthesia (1.5% in a 20% O_2_/80% air mixture) was acquired. For each rat, MRI was performed at 3 time points, with reference to V-COOL application: 24 h before V-COOL application, immediately after a 60-min application of the V-COOL device, 24 h after a 60-min application of the V-COOL device. Volumetric analysis of the entire ventricular system was quantified using MIPAV (NIH Center for Information Technology, Bethesda, MD, USA) and expressed in cubic millimeter.

### Statistical Analysis

Results were analyzed using SPSS Statistics (IBM Corporation, USA). Values were expressed as mean ± standard deviation. The three-group analyses on cortical temperature, rectal temperature, and intracranial pressure were done by means of one-way ANOVA. The two-group analyses on mortality, Garcia neuroscore, and corner turning test were done by means of Fisher’s exact test for dichotomized values and Mann–Whitney *U* test for ordinal and continuous values. A *p* value of less than 0.05 was considered significant.

## Results

### Analytical Modelling and Feasibility Study

**Fig. 2:**
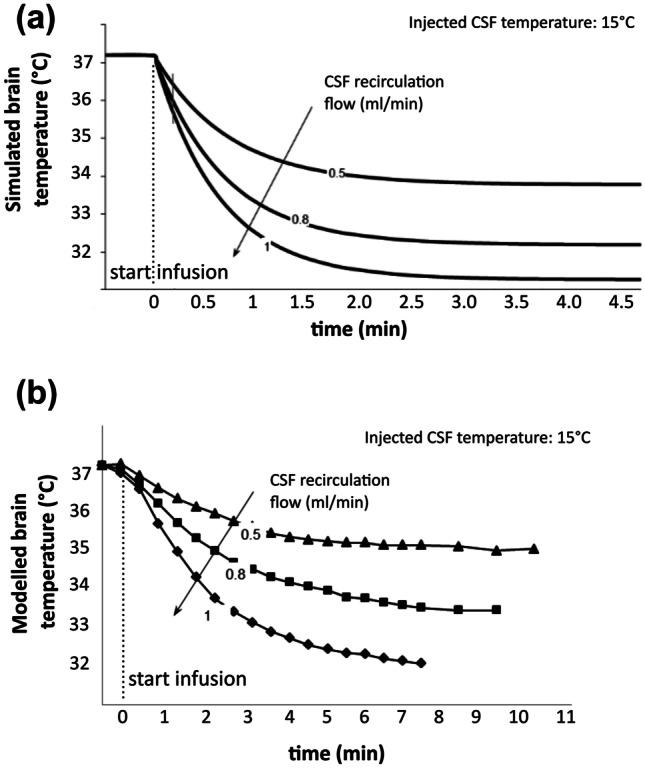
Thermometric curves related to inflow rates from in silico (**a**) and in vitro (**b**) modelling of the V-COOL device

A preliminary feasibility study of the approach was performed, using the simplified analytical model of brain-CSF-blood thermal balance, to estimate the degree and timing of cerebral parenchymal cooling after V-COOL application. Considering a temperature of 15 °C of the inflow CSF, a flow-dependent reduction of cerebral temperature (− 3.0 to − 5.8 °C) was obtained for inflow rates ranging from 0.5 to 1.0 mL/min, reaching a steady state within 5 min (Fig. 2a). In vitro experiments, using the simplified model of brain-CSF-blood interface within spherical PMMA capsule, replicated experimentally the same conditions of the analytical model. The in vitro model of V-COOL confirmed a flow-dependent reduction of cerebral temperature for inflow rates ranging from 0.5 to 1.0 mL/min, although a less efficient cooling was observed (from − 1.7 to − 4.5 °C), owing to the expected heat transfer. The dynamics of cerebral cooling of the in vitro model, however, was similar to the analytical model and reached a steady state within approximately 5 min (Fig. 2b). Overall, the analytical and in vitro preliminary analyses demonstrated the feasibility of the V-COOL prototype.

### In Vivo Efficacy of V-COOL: Effect on Brain and Systemic Temperature

**Fig. 3:**
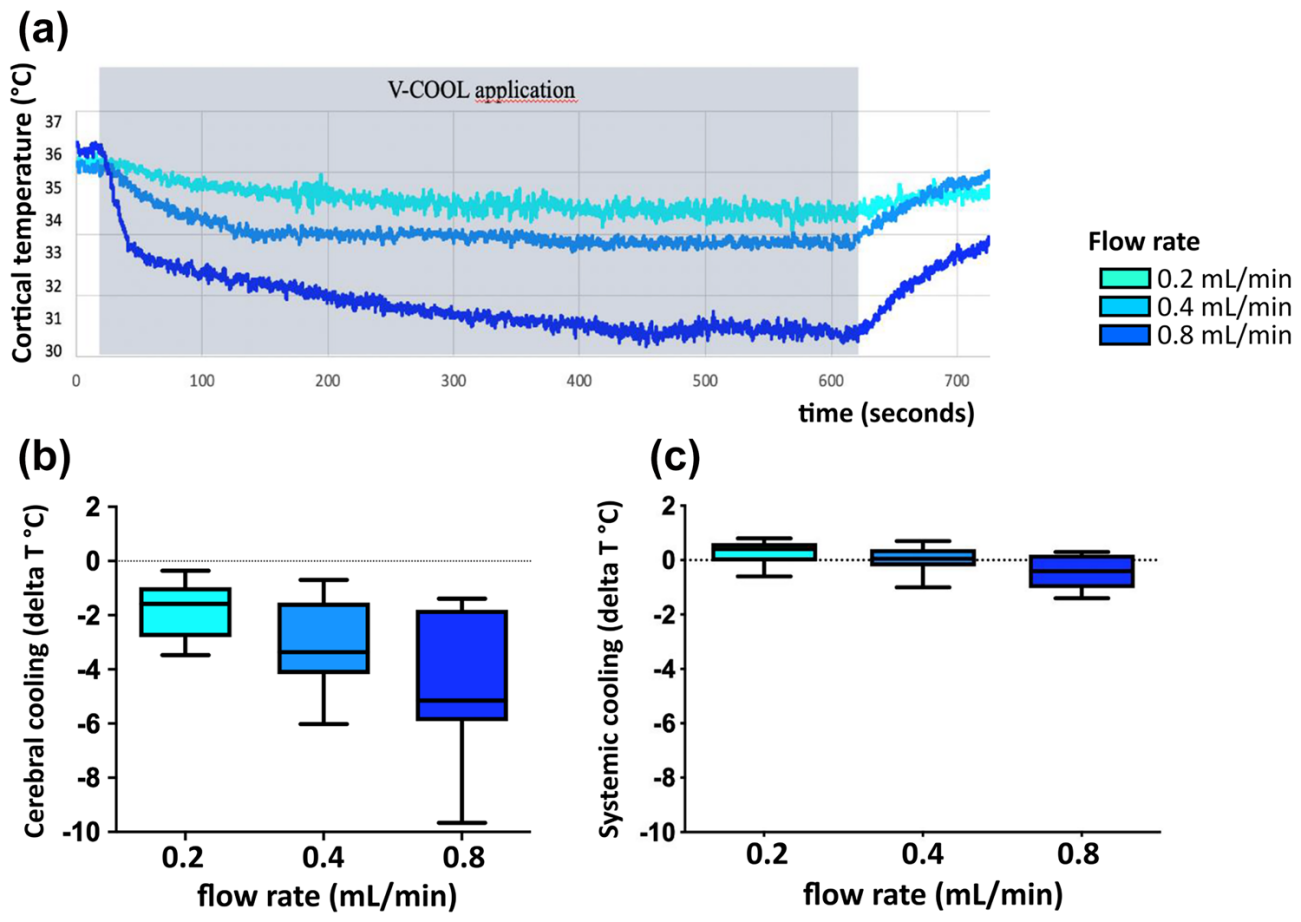
Representative tracings showing the dynamics of cerebral cortical temperature in a rat during application of the V-COOL device (**a**). Mean cerebral cortical cooling (*n* = 42) during V-COOL application at increasing inflow rates (**b**). Mean systemic cooling (rectal temperature, *n* = 42) during V-COOL application at increasing inflow rates (**c**)

In vivo thermographic monitoring was performed in 42 healthy rats during V-COOL application. The target degree of cerebral cooling was set to − 3.0 °C. Considering heat loss due to both thermodynamic and fluid dynamic shunt effect occurring in vivo, aCSF inflow temperature was set at 10 °C. According to the results of the in vitro model, inflow rates ranging from 0.2 to 0.8 mL/min were chosen to maximize both efficacy and safety. Duration of application ranged from 10 to 60 min. Application of V-COOL induced a dose-dependent mean reduction of brain cortical temperature in the ipsilateral hemisphere of 1.76 °C, 3.09 °C, and 4.46 °C at the flow rates of 0.2 mL/min, 0.4 mL/min, and 0.8 mL/min (*p* = 0.0008), respectively, with a time to steady state of 4.8 min (Fig. 3a, b). Rectal temperature was minimally affected by V-COOL application, with no change at 0.2 mL/min and 0.4 mL/min and a 0.4 °C reduction at 0.8 mL/min (*p* = 0.009; Fig. 3c). Preliminary experiments (*n* = 4) suggested a larger cooling effect in the ipsilateral hemisphere, compared to the contralateral hemisphere (Supplementary Fig. 1).

### In Vivo Safety of V-COOL: Effect on ICP and Functional Outcome

**Fig. 4:**
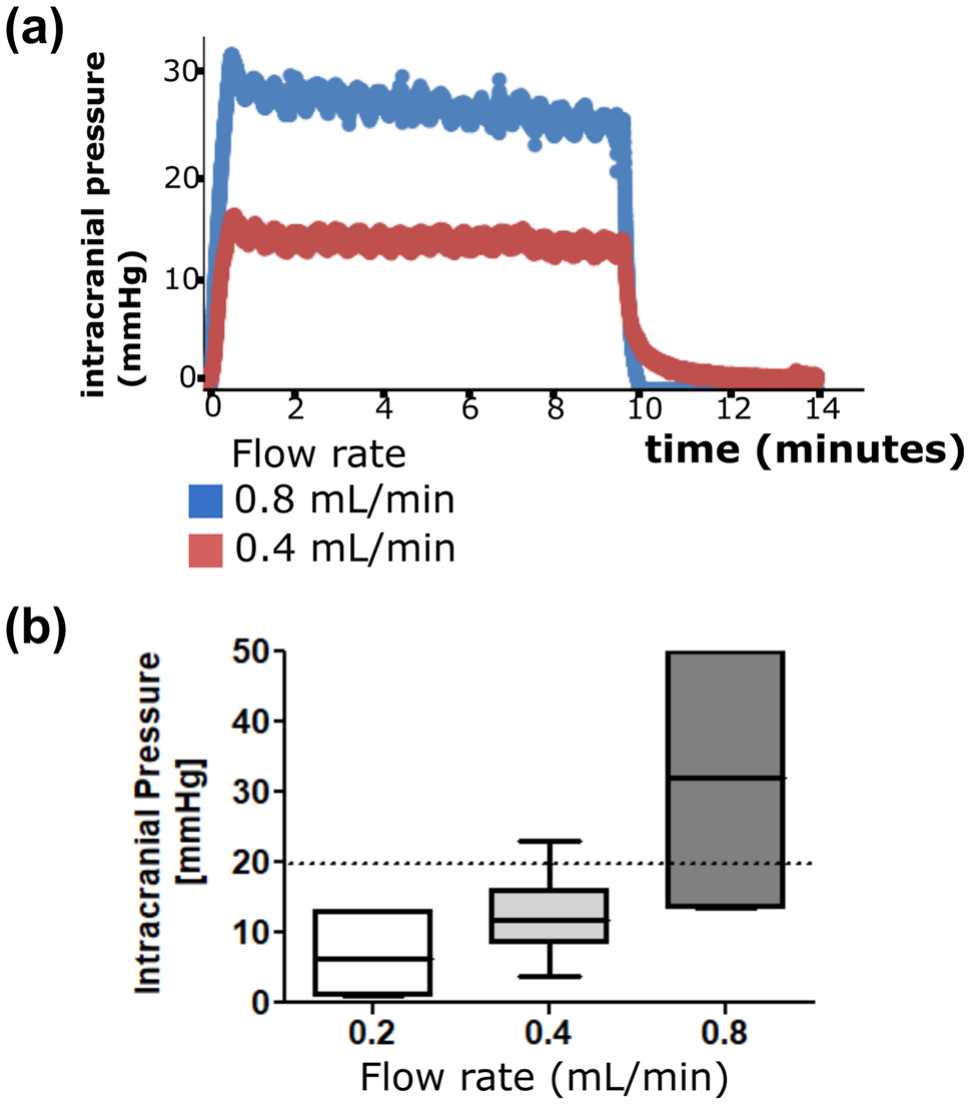
Representative tracings showing intracranial pressure changes in a rat during application of the V-COOL device (**a**). Mean intracranial pressure (*n* = 15) during V-COOL application at increasing inflow rates (**b**)

In vivo ICP monitoring was performed in 15 healthy rats. Before V-COOL application, baseline ICP was 5.9 (± 2.1) mmHg. After V-COOL insertion, the passive outflow in the extracranial reservoir was opened and ICP reached pressure equilibrium at 0 mmHg. After V-COOL activation, mean ICP values were 6.8 mmHg, 12.1 mmHg, and 31.8 mmHg at the flow rates of 0.2 mL/min, 0.4 mL/min, and 0.8 mL/min, respectively (*p* = 0.0002; Fig. 4a, b), reaching a plateau within 2 min. The flow rate of 0.4 mL/min was judged to have a favorable balance between cooling efficacy and ICP safety and it was chosen to be tested for 60-min application of V-COOL and assessment of imaging and functional outcome over the next 24 h. Functional outcome after 60 min of V-COOL application was assessed at 24 h and compared to sham surgery (Table 1). Neurobehavioral tests, comparing V-COOL application (*n* = 10) to sham surgery (*n* = 7), showed no difference in mortality (all animals survived), in the corner turning test (*p* = 1.000) and in the Garcia neuroscore (*p* = 0.485).

**Table 1:** Functional outcome 24 h after 60-min application of V-COOL

Outcome	Groups	Success, *n* (%)	*p* value	Scores (mean ± SD)	*p* value
Mortality	V-COOL	10 (100)	1.000	–	–
	Sham surgery	7 (100)			
Garcia neuroscore	V-COOL	8 (80)	0.485	15.1 ± 2.4	0.610
	Sham surgery	7 (100)		15.3 ± 0.3	
Corner turning test	V-COOL	9 (90.0)	1.000	48.8 ± 12.6	0.638
	Sham surgery	6 (85.7)		42.5 ± 21.2	

“Success” indicates a good functional outcome at 24 h (alive; Garcia neuroscore 14 to 18; % of right turns > 30%). “Scores” indicates ordinal data (3 to 18) for Garcia neuroscore and continuous data (0 to 100%) for corner turning test

*SD* standard deviation

### In Vivo MRI-Based Analysis of the Ventricular System During V-COOL Application

**Fig. 5:**
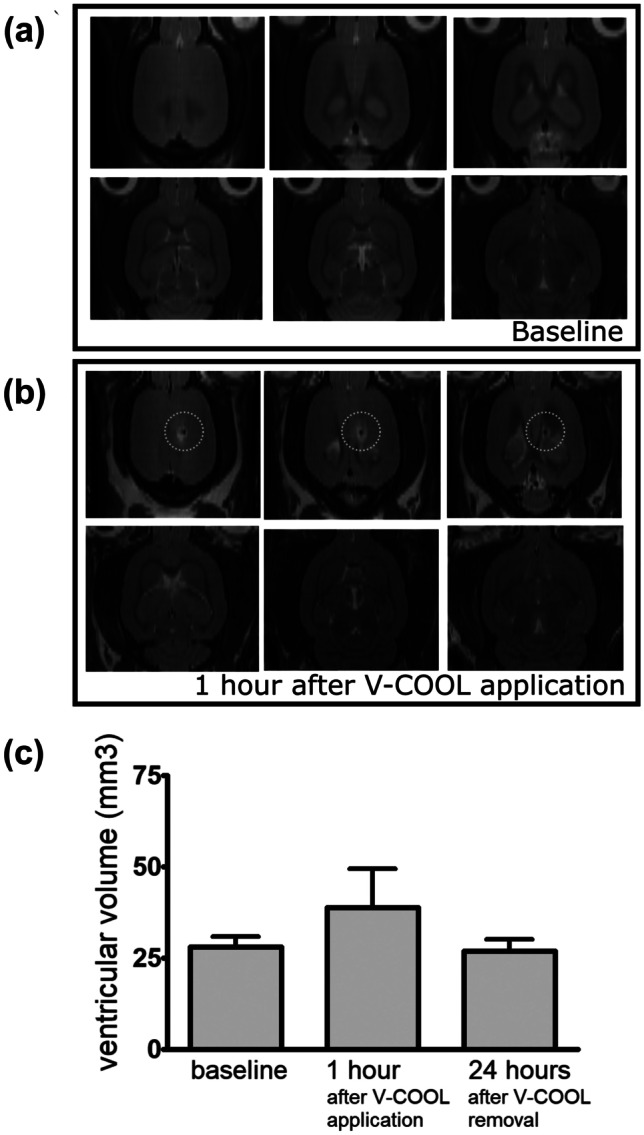
Representative brain MRI images (axial view) of a rat before (**a**) and after 60-min application of V-COOL (**b**). The site of V-COOL access is highlighted (dotted circles). Mean ventricular volume (*n* = 5) was calculated before, immediately after 60-min application of V-COOL, and 24 h after V-COOL removal (**c**)

The effect of 60-min application of V-COOL on the volume of the ventricular system was studied using MRI in 5 healthy rats, comparing baseline, immediately after 60-min application of V-COOL and 24 h after V-COOL removal. After 1 h of V-COOL application, ventricular volume increased by a mean of 38%, returning to normal values 24 h after V-COOL removal (Fig. 5a–c).

## Discussion

Mild therapeutic hypothermia (33–34 °C) has been shown to improve neurologic outcome in global cerebral ischemic conditions such as moderate-severity post-cardiac arrest syndrome in adults [15], hypoxic-ischemic encephalopathy in newborn infants [16], and aortic arch surgery [17]. A benefit of therapeutic hypothermia in focal cerebral ischemic conditions has been largely reported in experimental models of ischemic stroke [18] and SAH-induced vasospasm [19], but clinical studies have been inconclusive so far [20, 21]. Notably, a number of technical factors and predictable adverse events limited clinical translation of systemic hypothermia to acute neurological and neurosurgical patients [22–24].

In the present study, we aimed to go beyond the current applications of therapeutic hypothermia, exploring the new concept of selective CSF hypothermia and developing a ventricular cooling device (V-COOL). The aim of the V-COOL device is to rapidly obtain and maintain a target CSF temperature in a lateral ventricle, generating a temperature gradient across the ventricular system and two cerebral hemispheres. The cooling mechanism of the V-COOL device is based on exchange of cool-versus-warm CSF, similarly to hemodialysis or plasma exchange.

Previous studies explored different external devices aimed at inducing a selective brain hypothermia, using an intranasal cooling vaporizer, a cooling helmet, and a cooling collar. Overall, cerebral cooling efficacy of these external cooling devices proved to be modest. RhinoChill, a cooling technology providing transnasal evaporative cooling, was studied in pigs and in cardiac arrest patients and provided a net cooling effect of − 1.7 °C in 60 min and reached the target tympanic temperature of 34 °C in 75–85 min, when coupled with surface cooling [25, 26]. A brain cooling helmet was studied in patients with severe stroke and traumatic brain injury and provided a net cooling effect of − 1.6 °C in 60 min and reached the target brain cooling on − 3 °C in 3.4 h [27]. Neuron guard, a flexible cooling collar, was studied in sheep and provided a net cooling effect of 0.6 °C in 60 min and approached − 2 °C at the steady state [28].

Our findings showed the feasibility, efficacy, and safety of CSF cooling using the V-COOL device, which induced a clinically meaningful and rapid brain selective hypothermia. A net cooling effect of − 3 °C of the cerebral cortex was obtained within 5 min. Our results showed a favorable safety profile of V-COOL, with a flow-dependent increase of ICP, which could be maintained < 20 mmHg with a flow rate of 0.4 mL/min and a passive iso-pressure outflow. Under these conditions, application of V-COOL for 60 min in healthy rats did not affect survival and neurobehavior at 24 h and induced a mild and reversible dilation of the ventricular system.

Our study has several limitations, particularly regarding clinical translation. At the current stage, our results should be regarded as a first, proof-of-principle, experimental in vivo evidence of intraventricular cooling. The V-COOL technology needs further developments not only before being applied to patients, but also before being applied to animal models of acute brain injury which requires manipulation, surgery, and awakening from anesthesia.

A first translational development will be a flexible double-lumen cannula to test the V-COOL device in awake animals and in animal disease models. The current version of the V-COOL device, developed to be used in rats, was provided with a rigid cannula and was activated exclusively under stereotaxic guidance, due to of small size of the rat cerebral ventricles. Further studies in a large animal model of acute brain injury, such as the middle cerebral artery occlusion model in pigs [29], would allow to develop a new version of the V-COOL technology with a larger, flexible, double-lumen cannula.

A second translational development will be designing the V-COOL device as a closed-loop circuit, allowing re-circulation of the subjects’ own CSF. A “prompting” artificial CSF will be needed only to fill the circuitry of the device, without continuous refilling/wasting.

A third translational development, closely related to the ones discussed above, will be increasing safety through an optimized control of ICP and ventricular volume. A larger double-lumen cannula, connected to a closed-loop circuit, with a downstream ICP-dependent outflow reservoir, will guarantee improved outflow, reducing the risk of ICP increase. Moreover, the V-COOL device could be clinically developed to function also as an external ventricular drain to remove excess CSF, regulate ventricular volume, and guarantee ICP control in patients. Ideally, V-COOL might be integrated in a multi-lumen bolt including an ICP sensor, a temperature sensor, and a CSF drain.

Further safety issues should be addressed using future translational versions of the V-COOL device in large animal models, before moving to clinical studies: in case of mispositioning, an alarm could be activated by combining real-time data from the upstream pressure sensor and the outflow pressure sensor; biomarkers of parenchymal, ependymal, or meningeal inflammation could be analyzed in the outflow reservoir to further investigate tolerability; the exchange CSF rate tested in the rat version of the V-COOL prototype (0.4 mL/min) corresponds to approximately 50 mL/min in humans, which is approximately 10% of the exchange rate of hemodialysis (500 mL/min). It is imperative to understand the physiologic effects of rapid cerebral cooling in an animal model of acute brain injury. Attention should be high to detect unexpected adverse effects, learning from the experience with cardiac arrest, where rapid systemic cooling with intravenous cold saline decreased the rate of return of a spontaneous circulation in patients with an initial shockable rhythm [30].

Finally, further experimental data are needed to study the regional distribution and dynamics of brain cooling associated with the V-COOL device.

## Conclusions

No intraventricular cooling device is available at present for clinical use. Our findings provide proof-of-concept, experimental evidence of the efficacy and safety of this therapeutic approach at a pre-clinical level. Further experimental and clinical studies are needed to translate the V-COOL technology as an adjunctive treatment of SAH-associated DCI and other acute ischemic and non-ischemic brain disorders.

## Supplementary Information

Below is the link to the electronic supplementary material.

Supplementary file1 (PDF 185 kb)

Supplementary file2 (PDF 185 kb)

Supplementary file3 (PDF 185 kb)

Supplementary file4 (PDF 185 kb)

Supplementary file5 (PDF 185 kb)

Supplementary file6 (PDF 185 kb)

Supplementary file7 (PDF 185 kb)

Supplementary file8 (PDF 185 kb)

Supplementary file9 (PDF 185 kb)

Supplementary file10 (PDF 185 kb)

Supplementary file11 (PDF 185 kb)

Supplementary file12 (PDF 185 kb)

Supplementary file13 (PDF 185 kb)

Supplementary file14 (PDF 185 kb)

Supplementary file15 (PDF 185 kb)

Supplementary file16 (PDF 185 kb)

Supplementary file17 (PDF 185 kb)

Supplementary file18 (PDF 185 kb)

Supplementary file19 (PDF 185 kb)

Supplementary file20 (PDF 185 kb)

Supplementary file21 (PDF 185 kb)

Supplementary file22 (PDF 185 kb)

Supplementary file23 (PDF 185 kb)

Supplementary file24 (PDF 185 kb)

Supplementary file25 (PDF 185 kb)

Supplementary file26 (PDF 185 kb)

Supplementary file27 (PDF 185 kb)

Supplementary file28 (PDF 275 kb)

## References

[1] Mistry EA, Mistry AM, Nakawah MO (2017). Mechanical thrombectomy outcomes with and without intravenous thrombolysis in stroke patients: a meta-analysis. Stroke.

[2] Macdonald RL (2014). Delayed neurological deterioration after subarachnoid haemorrhage. Nat Rev Neurol.

[3] Conlon N, Grocott HP, Mackensen GB (2008). Neuroprotection during cardiac surgery. Expert Rev Cardiovasc Ther.

[4] Pilato F, Distefano M, Calandrelli R (2020). Posterior reversible encephalopathy syndrome and reversible cerebral vasoconstriction syndrome: clinical and radiological considerations. Front Neurol.

[5] Dumitrascu OM, Lamb J, Lyden PD (2016). Still cooling after all these years: meta-analysis of pre-clinical trials of therapeutic hypothermia for acute ischemic stroke. J Cereb Blood Flow Metab.

[6] Wassink G, Davidson JO, Lear CA (2018). A working model for hypothermic neuroprotection. J Physiol.

[7] Han Z, Liu X, Luo Y (2015). Therapeutic hypothermia for stroke: where to go?. Exp Neurol.

[8] Lewis SR, Evans DJ, Butler AR (2017). Hypothermia for traumatic brain injury. Cochrane Database Syst Rev.

[9] Zeiler FA, Zeiler KJ, Teitelbaum J (2015). Therapeutic hypothermia for refractory status epilepticus. Can J Neurol Sci.

[10] Riker RR, Gagnon DJ, May T (2015). Analgesia, sedation, and neuromuscular blockade during targeted temperature management after cardiac arrest. Best Pract Res Clin Anaesthesiol.

[11] Polderman KH, Herold I (2009). Therapeutic hypothermia and controlled normothermia in the intensive care unit: practical considerations, side effects, and cooling methods. Crit Care Med.

[12] Preparation of artificial CSF. In: Alzet osmotic pumps [online]. Available at: www.alzet.com/guide-to-use/preparation-of-artificial-csf/. Accessed 18 Sept 2021.

[13] Garcia JH, Wagner S, Liu KF (1995). Neurological deficit and extent of neuronal necrosis attributable to middle cerebral artery occlusion in rats. Statistical validation. Stroke.

[14] Krafft PR, McBride DW, Lekic T (2014). Correlation between subacute sensorimotor deficits and brain edema in two mouse models of intracerebral hemorrhage. Behav Brain Res.

[15] Nishikimi M, Ogura T, Nishida K (2021). Outcome related to level of targeted temperature management in postcardiac arrest syndrome of low, moderate, and high severities: a nationwide multicenter prospective registry. Crit Care Med.

[16] Kattwinkel J, Perlman JM, Aziz K (2010). Neonatal resuscitation: 2010 American Heart Association guidelines for cardiopulmonary resuscitation and emergency cardiovascular care. Pediatrics.

[17] Qu JZ, Kao LW, Smith JE (2021). Brain protection in aortic arch surgery: an evolving field. J Cardiothorac Vasc Anesth.

[18] van der Worp HB, Sena ES, Donnan GA (2007). Hypothermia in animal models of acute ischaemic stroke: a systematic review and meta-analysis. Brain.

[19] Torok E, Klopotowski M, Trabold R (2009). Mild hypothermia (33 degrees C) reduces intracranial hypertension and improves functional outcome after subarachnoid hemorrhage in rats. Neurosurgery.

[20] Geurts M, Petersson J, Brizzi M (2017). COOLIST (Cooling for Ischemic Stroke Trial): a multicenter, open, randomized, phase II, clinical trial. Stroke.

[21] Karamatsu JB, Kollmar R, Gerner ST (2015). Is hypothermia helpful in severe subarachnoid hemorrhage? An exploratory study on macro vascular spasm, delayed cerebral infarction and functional outcome after prolonged hypothermia. Cerebrovasc Dis.

[22] van der Worp HB, Macleod MR, Bath PM (2019). Therapeutic hypothermia for acute ischaemic stroke. Results of a European multicentre, randomised, phase III clinical trial. Eur Stroke J.

[23] Kuczynski AM, Marzoughi S, Al Sultan AS (2020). Therapeutic hypothermia in acute ischemic stroke – a systematic review and meta-analysis. Curr Neurol Neurosci Rep.

[24] Bohl MA, Martirosyan NL, Killeen ZW (2018). The history of therapeutic hypothermia and its use in neurosurgery. J Neurosurg.

[25] Islam S, Hampton-Till J, Watson N (2015). Early targeted brain COOLing in the cardiac CATHeterisation laboratory following cardiac arrest (COOLCATH). Resuscitation.

[26] Castrén M, Nordberg P, Svensson L (2010). Intra-arrest transnasal evaporative cooling: a randomized, prehospital, multicenter study (PRINCE: Pre-ROSC IntraNasal Cooling Effectiveness). Circulation.

[27] Wang H, Olivero W, Lanzino G (2004). Rapid and selective cerebral hypothermia achieved using a cooling helmet. J Neurosurg.

[28] Giuliani E, Magnoni S, Fei M (2016). A novel cooling device for targeted brain temperature control and therapeutic hypothermia: feasibility study in an animal model. Neurocrit Care.

[29] Arikan F, Martínez-Valverde T, Sánchez-Guerrero Á (2017). Malignant infarction of the middle cerebral artery in a porcine model. A pilot study. PLoS ONE.

[30] Bernard SA, Smith K, Finn J (2016). Induction of therapeutic hypothermia during out-of-hospital cardiac arrest using a rapid infusion of cold saline: the RINSE trial (Rapid Infusion of Cold Normal Saline). Circulation.

