# Supplementary figures and images for: Selective Cerebrospinal Fluid Hypothermia: Bioengineering Development and In Vivo Study of an Intraventricular Cooling Device (V-COOL)
Source: Neurotherapeutics. 2022 Sep 21;19(6):1942–50. doi: 10.1007/s13311-022-01302-y (PMC9723013; doi:10.1007/s13311-022-01302-y)

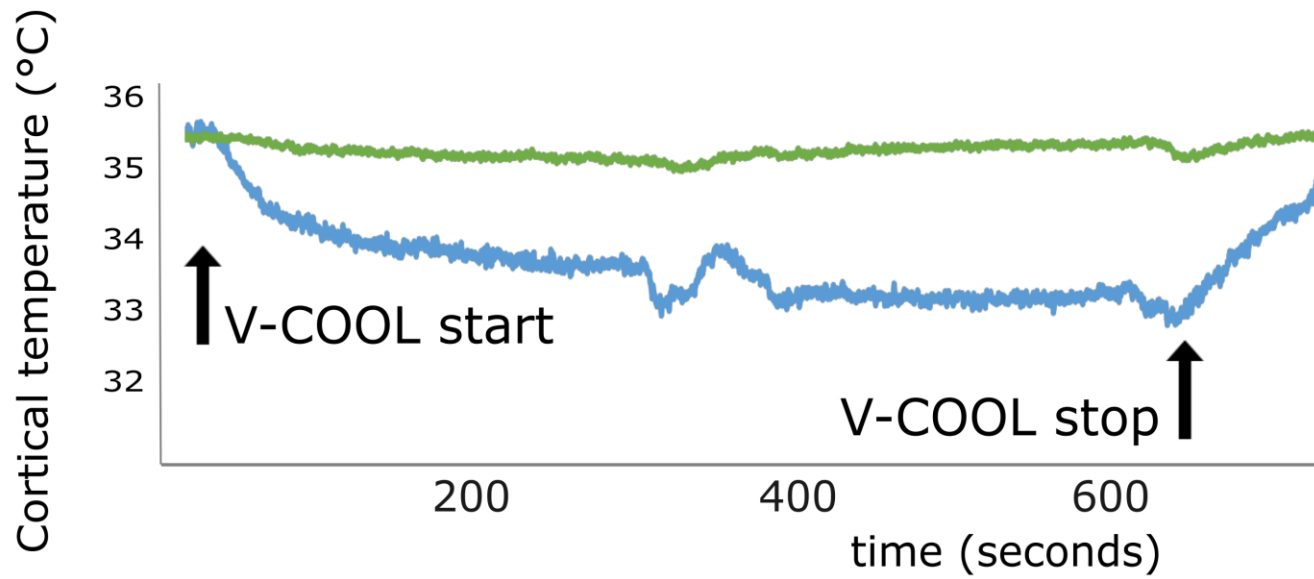

**Temperature  
probes**

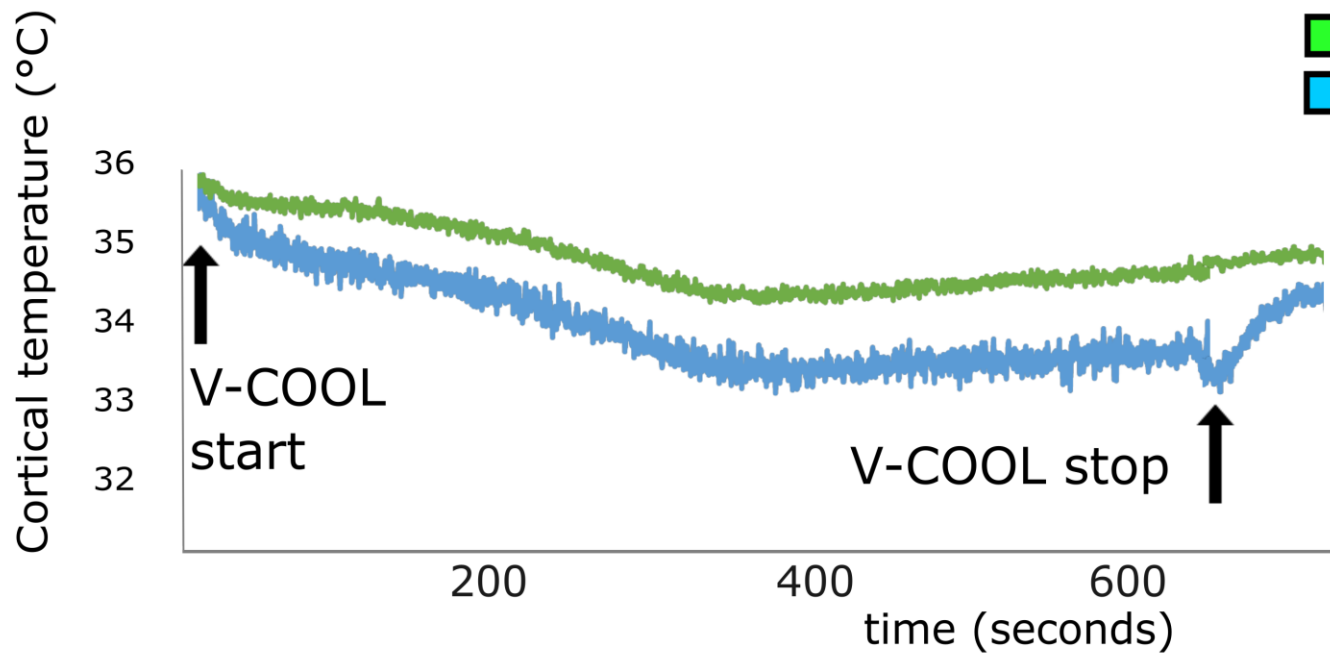

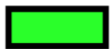 contralateral  
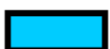 ipsilateral

Supplement: Supplementary file 28 — Supplementary file28 (PDF 275 kb) [file 13311_2022_1302_MOESM28_ESM.pdf]
